# APP Gene-based Strategies to Combat Alzheimer's Disease in Down Syndrome

**DOI:** 10.2174/0113892029360836250127103637

**Published:** 2025-02-03

**Authors:** Xu-Qiao Chen

**Affiliations:** 1 Department of Neurosciences, University of California San Diego, La Jolla, CA, USA

## 1 INTRODUCTION

Down syndrome (DS) is a genetic disorder caused by an extra chromosome 21 (HSA21). While medical advancements have extended the life expectancy of individuals with DS, they face a significantly higher risk of developing Alzheimer's disease (AD) as they age [1]. AD is a major factor limiting their lifespan, making it crucial to develop targeted therapies to delay or prevent its onset.

The β-amyloid precursor protein (APP) undergoes sequentially cleavage by β-secretase (BACE) and γ-secretase to generate β-amyloid (Aβ), with Aβ40 and Aβ42 aggregates accumulating in the brains of individuals with AD. Variations in the APP gene sequence or expression can modulate Aβ levels and composition, contributing to disease risk [2]. The *APP* gene, located on HSA21, encodes APP. Research indicates that the increased dosage of the *APP* gene in DS, as observed in cases of partial trisomy and familial AD, plays a critical role in AD development [3]. The increased *APP* gene dosage in DS leads to increased APP protein levels and higher concentrations of these toxic Aβ species, contributing to AD pathology in DS. This underscores the rationale for targeting APP as a therapeutical strategy to address AD in individuals with DS.

Addressing the effects of increased *APP* gene dosage involves multiple strategies, including reducing mRNA transcription, inhibiting protein translation, modulating APP processing, and enhancing APP clearance. Developing therapies targeting these mechanisms could help mitigate the impact of AD in individuals with DS.

**Fig. (1):**
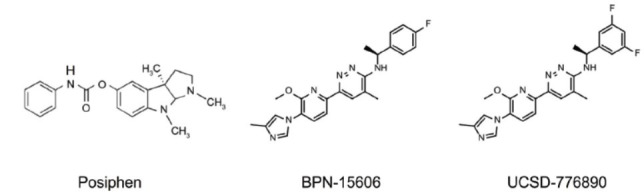
Structure of major compounds discussed in this editorial.

Inhibiting APP translation is a strategy that must be carefully considered due to the critical role of APP in physiological processes. This approach may be particularly relevant in cases of AD related to *APP* gene duplication and DS, where the *APP* gene is located on HSA21. One promising compound, posiphen (Fig. **1**), is an orally available small molecule that increases the affinity of iron regulatory proteins for the iron-response element. This increased binding reduces the translation of *APP* mRNA, which has been shown to alleviate pathological features, such as tau hyperphosphorylation and deficits in neurotrophin signaling in the Ts65Dn mouse model, which show segmental trisomy involving 90 mouse genes that have counterparts on the long arm of HSA21 [4]. Expanding on this method, additional APP translation inhibitors have been identified that specifically decrease the production of neural APP and Aβ [5]. These molecules have demonstrated improved potency and selectivity in cell models, although their efficacy and safety have yet to be tested *in vivo*. A recent report on a multicenter, double-blind phase 1b trial demonstrated that posiphen was safe and well-tolerated in individuals with early AD. However, the study involved a small sample size [6].

Antisense oligonucleotides (ASO) based strategies have also shown promise in reducing toxic proteins in neurodegenerative diseases. Compared to translation blockers, mRNA-based ASOs are more selective. For example, an ASO designed to promote APP exon 17 skipping generates an APP isoform lacking the γ-secretase cleavage site, thereby preventing Aβ peptide production. This ASO reduced full-length APP and Aβ42 levels in fibroblasts derived from DS patients [7]. A recent study further demonstrated that an APP-targeting ASO could reduce full-length APP and various Aβ species while also rescuing endolysosomal dysfunction in neurons derived from both APP duplication and trisomy 21 human iPSCs [8]. While these ASO-based strategies have shown potential in lowering APP and Aβ *in vitro*, their feasibility *in vivo*, particularly regarding potency, selectivity, and safety, requires further investigation.

Modulating APP processing by inhibiting BACE1 or γ-secretase to reduce Aβ levels, particularly the toxic Aβ42, is a logical approach to treating AD. However, many clinical trials using these strategies have been discontinued due to challenges with effectiveness and various side effects. A major issue is that both BACE1 and γ-secretase have multiple substrates beyond APP, leading to a lack of selectivity and resulting in unintended consequences and off-target effects, which may unpredictably impact neuronal and cognitive functions [9].

To address these challenges, γ-secretase modulators (GSMs) have been developed to selectively modulate this enzyme’s activity. GSMs aim to enhance the processivity of γ-secretase, thereby reducing the production of longer, more aggregation-prone Aβ peptides (such as Aβ42 and Aβ40) without significantly affecting their total levels or processing other substrates. This approach seeks to achieve a selective reduction of Aβ42 and Aβ40 while minimizing side effects. Earlier generations of GSMs, including non-steroidal anti-inflammatory drugs and heterocyclic compounds, suffered from weak potency, poor brain penetration, and various side effects. However, recent advancements have led to the synthesis of a novel and potent class of bridged heterocyclic compounds [9].

BPN15606 and UCSD-776890 (Fig. **1**), two highly promising γ-secretase modulators (GSMs), have undergone extensive preclinical testing in mouse models of AD and DS, as well as in nonhuman primate studies [10-13]. Preclinical studies have shown that these GSMs can reduce Aβ40 and Aβ42 without altering total Aβ levels. They also reduce amyloid plaques, tau pathology, astrogliosis, microgliosis, and reverse memory-linked behavioral deficits in AD mouse models [10-12]. Moreover, BPN15606 significantly decreases levels of Aβ40 and Aβ42 in the Ts65Dn mice. Additional benefits include rescuing the hyperactivation of Rab5, normalizing neurotrophin signaling deficits, and correcting abnormalities in synaptic proteins and tau phosphorylation. It also reduces astrocytosis and microgliosis and mitigates cognitive deficits in Ts65Dn mice [13]. Overall, these advancements underscore the potential of GSMs to selectively modulate APP processing and offer a promising approach to reducing Aβ-related pathology in DS-related AD.

In recent years, several anti-amyloid monoclonal antibody drugs targeting Aβ, including aducanumab, lecanemab, and donanemab, have been approved, further supporting the amyloid hypothesis. These antibodies bind to Aβ species, promote their clearance, and may potentially slow the progression of AD [14]. However, despite the high risk of AD in the DS population, clinical trials of these amyloid monoclonal antibodies have not yet included DS patients. It remains uncertain whether these drugs can effectively combat AD in individuals with DS. Hopefully, clinical trials in this population will be initiated soon.

A related study using vaccine-induced antibodies in Ts65Dn mice showed that these antibodies reacted with Aβ without detectable binding to either APP or its C-terminal fragments. Vaccination of Ts65Dn mice reduced brain Aβ levels to the levels in diploid (2N) mice. Importantly, vaccinated Ts65Dn mice showed improved memory and reduced cholinergic neuron atrophy [15]. These findings suggest that an anti-Aβ immunotherapeutic approach may effectively target Aβ-related pathology in DS mice. In line with this, the anti-amyloid ACI-24 vaccine was found to be safe, well-tolerated, and capable of triggering an immune response in adults with DS during a phase 1b trial [16].

## CONCLUSION

Several key factors must be considered when targeting APP in DS to address AD-related traits. While these strategies have shown significant potential *in vitro* and/or in animal models, further research is needed to evaluate their potency, optimal dosage, and safety in reducing APP and toxic Aβ species. Additionally, it is essential to determine whether these approaches can effectively reverse the multiple AD-linked phenotypes in DS *in vivo* from both prevention and treatment perspectives. As these strategies are being or will be tested in clinical trials, primarily in the AD population rather than directly in individuals with DS, these trials will provide important data on long-term safety, adverse effects, and therapeutic effectiveness, offering valuable insights into the potential applicability of these strategies to DS. Furthermore, genetic and environmental variations contribute to heterogeneity among individuals with DS. Developing personalized medical strategies tailored to individual characteristics is a necessary and long-term objective. Achieving maximum effectiveness requires a multidisciplinary approach that integrates neurology, genetics, psychology, and social sciences expertise to develop the most precise and personalized treatment strategies.

## LIST OF ABBREVIATIONS

APP: β-amyloid Precursor Protein
ASO: Antisense Oligonucleotides
Aβ: β-amyloid
DS: Down Syndrome
GSMs: γ-secretase Modulators
